# A Structured and Methodological Review on Vision-Based Hand Gesture Recognition System

**DOI:** 10.3390/jimaging8060153

**Published:** 2022-05-26

**Authors:** Fahmid Al Farid, Noramiza Hashim, Junaidi Abdullah, Md Roman Bhuiyan, Wan Noor Shahida Mohd Isa, Jia Uddin, Mohammad Ahsanul Haque, Mohd Nizam Husen

**Affiliations:** 1 Faculty of Computing and Informatics, Multimedia University, Persiaran Multimedia, Cyberjaya 63100, Malaysia; noramiza.hashim@mmu.edu.my (N.H.); junaidi.abdullah@mmu.edu.my (J.A.); wan.noorshahida.isa@mmu.edu.my (W.N.S.M.I.); 2 Technology Studies Department, Endicott College, Woosong University, Daejeon 32820, Korea; jia.uddin@wsu.ac.kr; 3 Department of Computer Science, Aarhus University, 9100 Aarhus, Denmark; iamahsanul@gmail.com; 4 Cybersecurity & Technological Convergence, Malaysian Institute of Information Technology Universiti Kuala Lumpur, Kuala Lumpur 50250, Malaysia; mnizam@unikl.edu.my

**Keywords:** gesture recognition, feature extraction, gesture classification, recognition accuracy, deep learning

## Abstract

Researchers have recently focused their attention on vision-based hand gesture recognition. However, due to several constraints, achieving an effective vision-driven hand gesture recognition system in real time has remained a challenge. This paper aims to uncover the limitations faced in image acquisition through the use of cameras, image segmentation and tracking, feature extraction, and gesture classification stages of vision-driven hand gesture recognition in various camera orientations. This paper looked at research on vision-based hand gesture recognition systems from 2012 to 2022. Its goal is to find areas that are getting better and those that need more work. We used specific keywords to find 108 articles in well-known online databases. In this article, we put together a collection of the most notable research works related to gesture recognition. We suggest different categories for gesture recognition-related research with subcategories to create a valuable resource in this domain. We summarize and analyze the methodologies in tabular form. After comparing similar types of methodologies in the gesture recognition field, we have drawn conclusions based on our findings. Our research also looked at how well the vision-based system recognized hand gestures in terms of recognition accuracy. There is a wide variation in identification accuracy, from 68% to 97%, with the average being 86.6 percent. The limitations considered comprise multiple text and interpretations of gestures and complex non-rigid hand characteristics. In comparison to current research, this paper is unique in that it discusses all types of gesture recognition techniques.

## 1. Introduction

### 1.1. Background

Hand gesture recognition plays a significant part in delivering diverse messages using hand gestures in the digital domain. Real-time hand gesture identification is now possible because of advancements in both imaging technology and image processing algorithmic frameworks. This has enabled natural interactivity previously unattainable by the use of the two-dimensional mouse. Due to the real-time nature of gesture recognition, it should be accomplished without overburdening the computing element. Moreover, image processing plays a critical role in segmentation, feature extraction of hand gestures and ultimate recognition of the gestures. Numerous computer vision algorithmic frameworks based on image processing concepts have been developed and are being improved.

Hand motions may vary from static to dynamic, depending on their use. Hand gesture recognition technologies each have their own set of benefits and drawbacks, which are dependent on the platforms on which they are implemented. Due to numerous difficulties encountered during foreground separation from the background, there are many current obstacles to achieving realistic and effective real-time hand gesture recognition. The hand that needs to be identified is represented by the foreground. Changing picture luminance, such as pixel color of the hand skin and background in vision-based systems, as well as cumbersome, expensive gear in glove-enabled and depth-enabled systems, are the most common problems.

### 1.2. Survey Methodology

The initial stage to the systemic literature review is to acquire all documents collected from 2012 to 2022, in which the screening procedure involves the download and lecture of the materials published in IEEE Xplore.

- Science, technology, or computer science are all acceptable search terms. Journals, proceedings, and transactions are the three main categories of publications.
- Article type is the in-depth analysis and commentary.
- The vision-based hand gesture recognition system can recognize a variety of different hand motions.
- The language of instruction is English.

**Figure 1:**
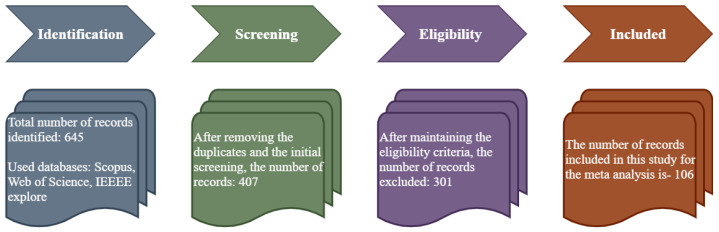
Block diagram of the article selection process.

The article selection process undertaken is illustrated in Figure 1. To identify relevant papers, we used three well-known databases: Scopus, Web of Science, and IEEE Xplore. We have also resorted to a number of reputable websites in order to obtain some particular information. The focus of this study is primarily determined by two keywords: hand gesture and computer vision. As a result, we utilized these two terms in conjunction with other keywords in the majority of our searches. Original articles, review articles, book chapters, conference papers, and lecture notes, among other kinds of materials, were gathered.

**Figure 2:**
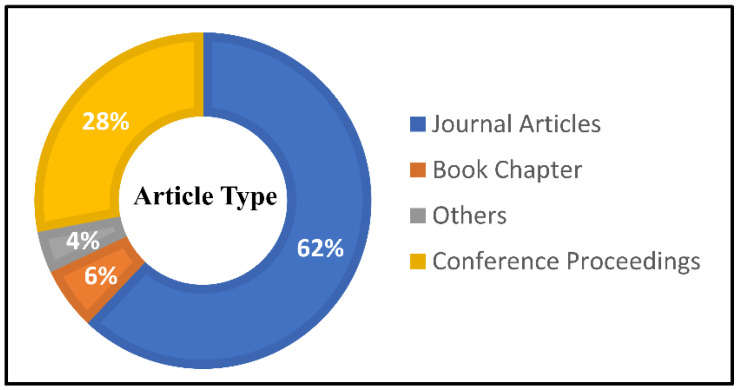
Journal articles, conference papers, book chapters, and other publications.

**Figure 3:**
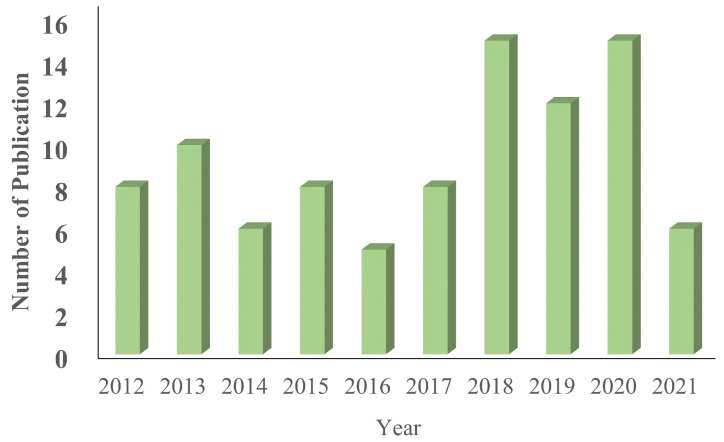
The number of peer-reviewed publications in relation to the year of publication.

We read the title, abstract, conclusion, and keywords in the first round of the review. Following the removal of duplicates, the surviving items are classified according to their intended use. During the first round of screening, hand gesture detection utilizing computer vision-based articles written in English are used as the selection criteria. The chosen papers are examined in the second stage of screening. The detailed approach serves as the ultimate criterion for article selection in this case. Only “108” articles are chosen for this evaluation after adhering to the aforementioned criteria. The number of chosen documents is divided into four categories: journal articles (62%), conference papers (28%), book chapters (6%), and others (4%), as shown in Figure 2. Figure 3 shows the number of publications of the chosen papers by year for this study. It may be argued that the greatest number of publications, 74, were chosen for this study during the past few years (after excluding irrelevant papers), indicating the significance of this area of research.

### 1.3. Research Gaps and New Research Challenges

This section summarizes the recognized research gaps as well as the forthcoming research issues. Hand gesture recognition systems have prospective uses, according to the study. Furthermore, the region has been proven to have a number of obstacles from three perspectives: system, environment, and gesture-related issues. The vast diversity of possible gestures is the primary difficulty in vision-driven gesture detection. The identification of gestures requires a wide range of degrees of freedom, as well as a large amount of 2D variability. Dealing with different degrees of freedom, huge variety in the look of 2D, given the camera perspective (having the same gesture), varied silhouette sizes (such as spatial resolution), and varying resolutions in the time dimension are all part of the gesture identification process (such as gesture speed variability). Regardless of the application type, solution cost, or other variables, scalability, robustness, and user independence must be balanced for the best balance of accuracy, performance, and effectiveness. This should be possible if the system is capable of evaluating incoming video frames and responding to recognized gestures in real time. The requirement for resilience is one of the most essential elements for robustness in the identification of various hand motions in various light and busy situations. It is also crucial that the system can rotate pictures in-plane and out-of-plane without breaking. Furthermore, scalability ensures that a large gesture vocabulary, consisting of just a few fundamental motions, can be handled. In this respect, the user has complete control over the composition of user gestures. Not only that, but the system independence encourages a collaborative work environment in which many users, rather than a single user, are in control, allowing for the more accurate detection of human gestures of various sizes and colors. Remember that each of these technological enablers has advantages and disadvantages. The requirement of physical contact for contact-enabled devices can be discomforting to the users; however, such devices provide high recognition accuracy with reduced complexity in terms of implementation. As much as vision-enabled devices are user-friendly, they face configuration complexity in addition to occlusion.

### 1.4. Contribution

This article reviews current developments in the field of human–computer interaction (HCI). The emphasis is on the different application areas, where hand gestures are used to create effective engagement. The goal of this article is to give an overview of the current status of static and dynamic hand gesture recognition in the area of HCI, including gesture taxonomies, representations, and recognition methods, as well as to identify future research objectives in the field.

### 1.5. Research Questions

The following major questions are addressed by this research:

1. What are the main difficulties faced in gesture recognition?
2. What are some challenges faced with gesture recognition?
3. What are the major algorithms involved in gesture recognition?

### 1.6. Organization of the Work

**Figure 4:**
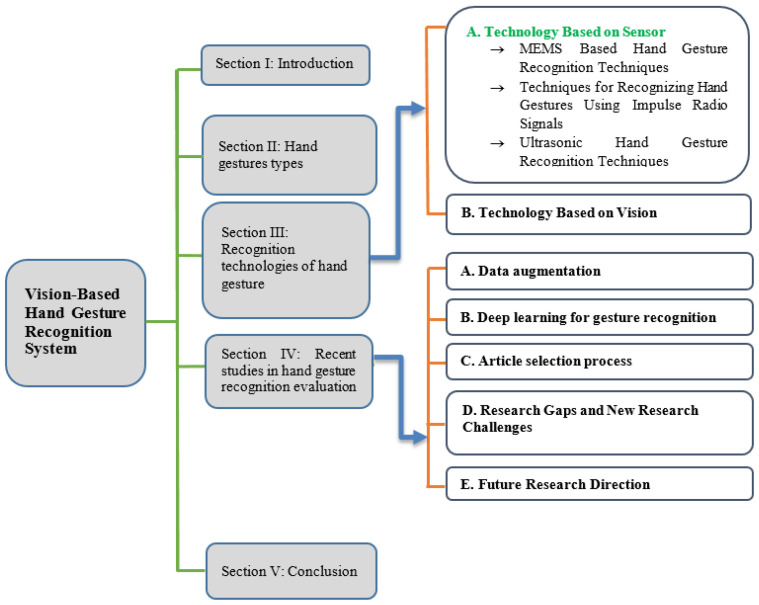
Five class graphical presentation.

The rest of the paper is organized as follows: Section 3 introduces the types of hand gestures. Section 4 considers an evaluation of the latest research works in regard to hand gesture recognition. Section 5 considers the current research challenges in this field. Lastly, Section 5 provides the conclusion. The overall organization of the work is nicely depicted in Figure 4.

## 2. Hand Gestures Types

Hand gestures are a kind of body language in which the position and shape of the center of the palm and the fingers communicate specific information. The gesture is made up of both static and dynamic hand movements in general. Dynamic hand gestures are made up of a series of hand movements, while static hand gestures are based only on the shape of the hand. Different individuals describe gestures differently due to the cultural variety and uniqueness of gestures. Static hand gestures rely on the shape of the hand gesture to convey the message, while dynamic hand gestures rely on the movement of the hands to transfer the meaning. The ability to instantly and without delay identify hand motions is known as the detection of real-time hand gestures. Processing speed, image processing techniques, acceptable delay in conveying results, and recognition algorithms differ between real-time and non-real-time hand gestures.

## 3. Recognition Technologies of Hand Gesture

**Figure 5:**
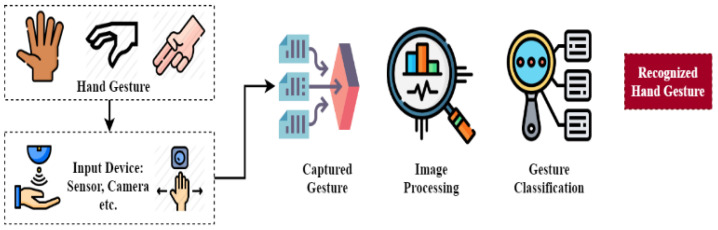
General framework about hand gesture recognition.

**Figure 6:**
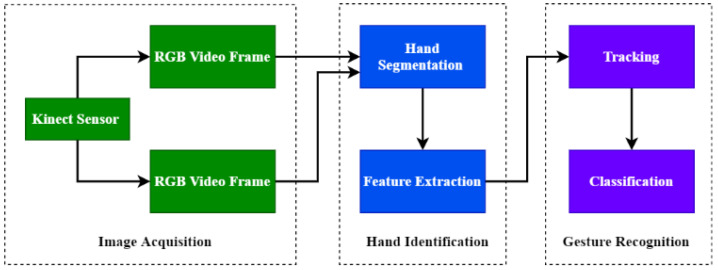
Standard framework for hand gesture recognition using Kinect.

The results of the research show that hand gestures allow technology to be divided into three types: sensor-driven, vision-driven and deep learning. Sensor-based technology, as its name suggests, uses different sensors such as the accelerometer and the gyroscope, while RGB cameras and infrared sensors are used to extract and identify properties from a collection of datasets of hand movements, respectively. The general framework about hand gesture recognition and standard framework for hand gesture recognition using Kinect is shown in Figure 5 and Figure 6, respectively.

### 3.1. Technology Based on Sensor

In sensor-based hand gesture recognition algorithms, motion sensors, which are either integrated into gloves or utilized in smart devices such as smartphones using built-in accelerometers with gyroscope sensors, are used.

The main objective is to gather and use triaxial data for the proper application. This method of gesture recognition in a smart gadget that uses a three-axis accelerometer and Gyro sensor, Ref. [1] presents a continuous hand gesture identification (CHG) technique to constantly identify hand movements. A Samsung ATIV smartphone is employed as an intelligent tool for gesture motions, and smart devices are operated using the Samsung AllShare protocol. Since the waveforms reflect change in amplitude and phase, the machine techniques such as CNN may be used for sensing them. Ref. [2] presented the feedforward neural network and similarity matching (FNN/SM) hand motion detection technique for accelerometer-based pen type sensing devices. A triaxal accelerometer, which is preprocessed by a moving average filter, is used to identify hand motion acceleration data. There is also a segmentation algorithm to control each key motion on the fly at the initial and final positions. Using the basic gesture samples used to train the FNN model after feature extraction, the fundamental gesture sequences are categorized. The series of basic acts is then coded using the codes of Johnson. Finally, the complex gesture is found by comparing the anticipated basic series of gestures with frequent template sequences.

#### 3.1.1. Techniques for Recognizing Hand Gestures Using Impulse Radio Signals

The transmission (Tx) produces an infrared signal through its antenna and sends it. The waved shape of the hand (Rx), consisting of an antenna, amplifiers, a low-pass filter and an oscilloscope with a high speed, is received from the receiver. Due to the varying amplitude and phase of the reflected waveforms, movement shapes may be used in machine learning methods, such as CNN. It is demonstrated in the picture below that this technique works.

#### 3.1.2. Ultrasonic Hand Gesture Recognition Techniques

In this technique, loudspeakers and microphones are utilized as ultrasonic I/O devices. The Doppler shift of ultrasonic waves reflected in a moving human body is used in this technique. During a gesture, the system constantly samples the ultrasound. It produces a series of time variations which are rich in the distinct characteristics of each action. We classify future motions using a mixture of fundamental patterns and supervised methods of machine learning.

### 3.2. Technology Based on Vision

The three essential phases of a vision-based method are image acquisition, image segmentation and lastly image identification. Many academics have developed hand motion detection systems based on these three stages in real time. The dynamic recognition of hand gestures is the identification of a moving hand with a number of motions, whereas the recognition of the hand position is static hand gestures. Hand motions may be visually categorized utilizing methods based on 3D modeling and looks. Those are the visual sub models of 3D models and model-based appearance methods. The 3D hand gesture model provides a 3D spatial representation of the human hand with time automation. The four kinds of appearance-based hand gesture representation methods are color, silhouette, decorative, and motion-based models.

Vision-based techniques for recognizing gestures: as previously mentioned, there are three basic stages: detection, tracking, and recognition. Ref. [3] explains briefly the numerous sub-techniques employed at various phases of their study. The main phases in the detecting phase include color, shape pixel, 3D model, and motion. Correlation-based and contour-based tracking are two kinds of template-based tracking. Other tracking techniques include optimum estimation, particle filtering, and camshift. Using a range of algorithms and machine learning techniques, the last stage in the complex identification process is to identify static and dynamic hand movements. Time delay neural networks, hidden Markov models, dynamic time warping networks, and finite state machines are examples of these techniques.

The most frequent use of color detection is the detection of skin color on the hand. The color space conversion to either HSV or YCbCr is done first for reliable hand segmentation. Binarization is achieved using skin color threshold values, and noise is reduced using image processing techniques, such as morphological processing. The segmented hand is then identified using a variety of techniques for extracting features and recognizing the segmented hand. One of the study articles by [4] was based on robust hand motion segmentation utilizing YCbCr color space and K-means clustering.

Tracking is essential since it sees and detects the hand in real time for the identification of hand gestures. Many tracking methods, one based on contour moments, were proposed. In order to perform hand center identification, ref. [5] utilized transformation and contour detection on complicated backdrops, which could be used for hand tracking. After that, the fingertip position method used to detect hand motions was calculated using a convex hull. In 1972, Sklansky launched the three-coin technique of the convex hull.

## 4. Significant Research Works on Hand Gesture Recognition

The focus in the preceding part was on the work’s initial components. This section builds on that basis by providing in-depth coverage of recent hand gesture recognition research studies that have been selected and reviewed. These works were carefully chosen and adapted to satisfy the challenging requirements in this field. Some of the works analyzed were clearly not evaluated in previous publications, implying that they are recent works. In the case of identity-based hand gestures, ref. [6] used a multivariate Gaussian distribution. The scientists divided the image into two procedures in their study: skin color-based segmentation and cluster-based thresholding. The restructuring of sign language is accomplished using a variety of approaches. Ref. [7] proposed a random forest-based wearable motion recording sensor for hand gesture detection. Gesture characteristics were gathered at various time intervals to quantify hand movements. The experimental evaluation is based on a dataset of gestures from a gesture pool. Ref. [8] enhanced gesture detection by employing the custom classifier in the frictionless operating room. A computer-aided surgical technique was proposed for the categorization of user movements. To forecast the result, vector support machines (SVM) and classifiers from naive Bayes were used.

Hand gesture recognition for healthcare was developed by [9]. To identify the hand motions without any noise, a convolutional neural network (CNN) was suggested. For segmentation, a MobiGesture was required to enhance accuracy and recall depending on user motions. Ref. [10] created a wearable sensor with a double surface EMG. Support vector machines were used to classify gesture recognition (SVM). Using two EMG channels on flexors and extensors, four types of gestures were identified.

The project’s purpose was to eliminate the gesture’s failure tolerance. An EMG armband was created by [11] to recognize human motions based on physiological characteristics. Wearing-independent hand gesture recognition has been developed. The EMG elements gave the gesture recognition a distinct scale. The uniform signal helped to enhance the recognition. A random forest was utilized to determine how gestures would progress and be pursued. Ref. [12] investigated dynamic hand movements using spatial–temporal algorithm convolutional networks. Three types of graph edges associated with the activity of hand joints were proposed in a skeleton-based model. A deep neural network was utilized to pick semantic characteristics in order to provide an accurate output. Ref. [13] created a string matching technique for understanding hand motions in real-time situations. The k-means technique was used to create an approximation string matching to capture the features of hand joints. It was done to enhance the precision of various motions by specifying the amount of clusters. Ref. [14] discovered maneuvers using the convolutional pose machine (CPM) and Gaussian mixing models of Zhang et al. (FGMM). The first stage was to acquire critical hand points with the CPM. The FGMM was then used to filter and categorize non-gestures by critical point. Ref. [15] developed a real-time fine gesture detection system using a convolution neural network. The system for monitoring muscle contraction in the forearms was built using the frequency–time–space cross-domain pre-processing method. Ref. [16] used jump motion with spatial fuzzy matching (SFM) to enhance hand gesture detection. The gesture matching was performed by analyzing the fused gesture dataset, where the gesture frames were categorized. The SFM was then utilized to accelerate the analysis processing.

To improve the efficiency of gesture analysis, ref. [17] created online dynamic hand gesture detection. The technique was selected for three reasons: there was no evidence of when the gesture began and ended; the rearrangement was only recognized once; and the gesture under investigation had to be within memory and budget. Gesture recognition and categorization are aided by the use of CNN to build a two-level hierarchy structure (TLHS).

Ref. [18] used long short-term memory(LSTM) to accomplish continuous hand gesture recognition. The analysis was used to detect input gestures using accelerators and/or gyroscopes. The output was acquired using LSTM, which was accomplished by accurately categorizing the generated gesture. Ref. [19] suggested a hand gesture approach to control powerpoint presentation. Primarily, the proposed approach can ensure the storage of gesture images without need for a database.

To up-scale the mouse capabilities, ref. [20] proposed a method based on the mouse pointing device. In addition to being a vision-based interface, the technique uses a camera. The solution can recognize a variety of predefined hand postures, including gesture mode and mouse mode revealing. The method uses the HSV color space to identify skin after obtaining the pictures.

A technique for guaranteeing the identification of gestures in real time was devised by [21]. A programmable field gate array was used to develop the technique. Ten distinct static hand movements may be recognized by the program. To represent the system, the authors used the verilog hardware description. Initially, the picture is acquired using a camera with a complementary metal oxide semiconductor image sensor during implementation. The image preparation is then completed. Finally, the system uses the YCbCr color space to conduct skin color segmentation on that picture. The 25 unique hand pictures are evaluated for gesture categorization, resulting in a 94.40 percent recognition rate. Ref. [22] examined the human–computer interaction from the standpoint of hand gesture recognition. The authors discussed past hand gesture detection efforts as well as the technological challenges they encountered. Vision-driven, glove-driven, and depth-driven techniques are all being explored. Finally, the study analyzes Microsoft’s release of Kinect data for finger and hand motion recognition.

To operate Power point and VLC media players, ref. [23] proposed a hand gesture detection method. Ref. [24] described vision studies conducted over the past 15 years that focused on identifying hand gesture methods. In addition, this study covers more than two dozen open-access hand gesture datasets, as well as download links. Ref. [25] proposed a framework for hand gesture recognition. The scientists looked at how they might utilize data from Kinect to recover depth information on all of the pixels in a image. They also developed a method for identifying the corresponding closed contour points in the desired depth intervals. The palm center is recognized right away from the recovery points. Furthermore, the fingers were detected using the k-curvature method. The first in, first out (FIFO) method was used to gather the contour points, palm center, and fingers throughout the procedure. After that, a DTW algorithm was used to identify gestures. Ref. [26] proposed the use of vision technologies to detect hand movements. To obtain the required outcomes, the authors used techniques, such as skin color filtering, edge detection, and convex hull. Ref. [27] looked at a technique for identifying hand gestures that utilizes 3D depth sensors. Three-dimensional hand modeling, static hand motion, and hand route gesture are among the work fields. The authors emphasized gesture recognition techniques in their work, as well as the regions that are utilized in such systems. Ref. [28] used a vision-driven recognition technique, as well as a library of hand gestures based on skin color architecture and threshold approaches through PCA template matching. The procedure starts with hand segmentation, which is done using a skin color model. The hand picture is then separated from the backdrop using the Otsu thresholding technique. Finally, the PCA employs a template-driven matching approach to accomplish gesture recognition. Ref. [29] considered a review of the Kinect-based markerless two-hand gesture detection method. The authors utilize Kinect’s depth and skeleton to accomplish marker-free hand extraction. The finger Earth mover’s distance (FEMD) is utilized for gesture measurement in the novel morphological finger extraction technique. Furthermore, for natural and reliable human–computer interaction, two hand-driven user interfaces are utilized. The collected results demonstrate the system’s efficacy.

Gesture communication was studied by [30]. Their research highlighted the importance of both nonverbal and vocal communication. Furthermore, they utilized nonverbal communication in their 3D game. Four stages are explored while developing a hand gesture recognition system. Data collection, segmentation, feature extraction, and gesture recognition are among these stages.

In this research, ref. [31] examined the importance of delivering a message from a user to a person through a man–machine interface. In the vision-driven approach, their work disregards and eliminates the usage of sensors linked to the human body. Furthermore, two important apps are used to operate the TV and mobile device via hand gestures. Through the use of gestures, the user can ably achieve direct communication with the system. The solution focused on the need to ensure successful user-friendly, as well as machine-friendly applications. The hand gesture selection module enables the user to customize the interface of TV control through selection of the shapes and hand motions.

Ref. [32] proposed an RGB-D sensor-based hand gesture detection method. The method utilizes deep knowledge to minimize light conditions and overwhelming background problems. The four stages of the method are hand segmentation, extraction of characteristics, static gesture classification and dynamic gesture classification. Segmentation of the hand is also carried out by segmenting the skin color, while background removal is utilized to segment the hand. The hand is removed afterwards. While fingertip detection is used to create static movements, dynamic gestures occur at the Euclidean distance. All this consists of 90 photos of 9 individuals with 10 dynamics and 6 motions. Ref. [33] proposed a hand gesture-based HCI approach. The system is built on vision-based technology and uses machine learning methods to accomplish its goals. Pre-processing, feature extraction, and recognition are the three stages of the technique. The system uses an ML classifier to achieve recognition. SVM is used to deal with static motions, whereas HMM is used to deal with dynamic gestures. The experimental findings indicate that employing 11 pre-defined motions, SVM and HMM achieved an accuracy rating of 99.7% and 93.7%, respectively.

For HCI, ref. [34] proposed a hand gesture detection system based on vision techniques. The authors developed a real-time application that uses a color-based detection method instead of ANN training to restrict mouse movement inside windows. Ref. [35] proposed a method for determining finger and hand posture based on depth information. To solve the problem of merging fingers, the method shows an apex-shaped hand structural contour. To accomplish hand identification, the authors also use depth thresholding. Using a dataset of 1000 postures, the findings show a 99.1% accuracy with FD and a 96.3 percent accuracy with global finger information. Ref. [36] suggested a depth sensor home-based hand gesture recognition method. The method is divided into two stages. The first step allows the creation of the required database. In the second step, the system extracts various features from the labeled hand parts to issue commands to the devices. Ref. [37] suggested a shape parameter based method. Moreover, to generate the shape information, a computer vision method was employed. Basically, the proposed hand gesture solution is matched through shape-enabled techniques. Ref. [38] recommended a novel approach for hand part segmentation based on the use of adaptive skin color architecture. Initially, the method grabs hand part pixel values and background part, thus changing them into the YCbCr color space architecture. Next, skin and Gaussian models are presented. Lastly, the approach performs segmentation. Ref. [39] used image and computer vision technologies to accomplish computer-aided control and monitor utilizing hand motions. Furthermore, the Haar classifier is used to guarantee face detection, and YCbCr is used to achieve hand gesture recognition. Hand tracking is done via the hand tracking technique, and features are extracted using the convexity defect hull. Furthermore, the hand region feature is controlled by the mouse position. Ref. [40] looked at how deep estimation and gesture recognition might be used. In terms of suggested techniques for hand recognition and gesture classifications, the authors focused on depth-based gesture identification. In the work, 13 methods, 11 of which were determined to be related to hand localization and gesture classification, in that order. Moreover, both the Kinect sensor and OpenNI were employed to ensure hand tracking. In total, 37 works were covered pertaining to the definition of gesture type and classification based on cluster depth. Ref. [24] conducted a review on the roles of the HCI system in the successful realization of hand gesture recognition. Their work shows that for current research, issues in hand gesture recognition include sensitivity in regard to size, shape speed variability and occlusion concerns. Their work focused on algorithms for vision-based hand gesture recognition. Moreover, they compared both quantitative and qualitative algorithms using RGB and RGB-D cameras. Additionally, several experimental simulation methodologies and measures were employed to evaluate the algorithms. Also reviewed are tens of publicly accessible hand gesture databases and the related links for download. Ref. [41] presents a useful idea for using mouse points to perform a variety of tasks, including single and double clicking, dragging, and so on. They claim that recognizing motions is challenging since many factors are involved, including modeling and movement, analysis, and pattern recognition. Ref. [42] developed a method to evaluate the dissimilarity of hand movements based on a super-pixel earth mover’s distance (SP-EMD). Furthermore, the texture and depth of the hand are conveyed via super-pixels, allowing the color of the motion to be recognized. It becomes invariant in terms of scaling, translation, and rotation after appropriate processing. The collected findings show that the identification speed is quick and the mean accuracy is high.

HCI was regarded by [22] to be an essential component of hand gesture recognition. They reviewed the history of hand gesture recognition and the technological challenges that come with it. The authors also explained the different methods, such as vision-based, glove-based, and depth-based ones. In addition, the authors discussed research projects using Microsoft’s Kinect device data, which includes novel finger identification and hand motion recognition. Finally, attention is given to Kinect-based applications, such as clinical surgery and robotics. Ref. [27] investigated the field of hand gesture detection using 3D depth sensors. The authors demonstrated that commercial depth sensors and publicly available datasets are widely used in the area of 3D. In addition, the systems’ different fields include 3D hand modeling, static gesture, hand trajectory gesture, and continuous hand gesture detection. Furthermore, the system makes use of cutting-edge research for 3D hand gesture detection. The work’s primary emphasis is on gesture recognition techniques and a clear description of the areas where this approach is used. Ref. [43] looked at using a vision-based technique to create a natural and intuitive interface. The authors concentrated on approaches for recognizing dynamic hand gestures. Their research is divided into three sections: detection and segmentation, tracking, and classification. The numerous segmentation methods based on skin color, shape, particle filtering, TLD, camshaft, and other factors are explained. In terms of applications, it has had a significant influence on daily life in a variety of ways.

The research of [44] is focused on gesture-based first-person control. The gamepad and a combination of keyboard and mouse are the two methods of input. The end product shows how to operate a first-person shooter game with a single hand gesture, followed by a comparison of standard input techniques [45]. Around 26 people collaborate to give the outcomes, which include summaries of inline performance and a survey of previous games [46]. The player’s abilities were developed using their outcomes [25,47]. There are designs in the scientific literature which are designed to identify and classify the hand gestures of static and dynamic types. These ideas are built on infrared pictures, color images and depths [48,49,50]. This study focuses on a literature review of hand gesture strategies and discusses their pros and limits in various situations. In addition, the performance of these methods is tabulated, with an emphasis on computer vision techniques that deal with similarity and difference points; hand segmentation techniques; classification algorithms and limitations; number and types of gestures; dataset used; detection range (distance); and camera type [51]. Convolutional neural networks (CNN) are used to categorize images of hand gestures. A newly developed metaheuristic technique, the Harris hawks optimization (HHO) algorithm, is utilized to optimize the CNN’s hyperparameters. Their extensive comparison research shows that the proposed HHO-CNN hybrid model outperforms current models by achieving 100 percent accuracy [52].

This article examines the flex, accelerometer, and gyroscope-based smart prototype developed to recognize sign language motions. These sensors are put on a glove in order to record and assemble alphabetic (i.e., 0–10, A–Z) and numeric (i.e., 0–10, and A–Z datasets). The primary purpose of the proposed model is to categorize sign gestures produced by deaf–mute people and identify the true meaning of movements performed [53].

Based on the ultrasonic frequency modulated continuous wave (FMCW) and the ConvLSTM model, a system for gesture identification was suggested in this work. It uses a hardware configuration consisting of one transmitter and three spatially separated receivers [54]. In this research, the authors proposed a hand gesture detection system for a dataset of lowercase numbers and alphabets. The suggested method recognizes the hand using information on skin color and mobility. Hand tracking is performed using a two-level tracking system and a modified Kanade–Lucas–Tomasi (KLT) tracking algorithm [55]. Table 1 presents the comparison of the most recent selected gesture recognition studies.

### 4.1. Data Augmentation

Data augmentation is a technique of expanding the data set by producing various picture shapes to increase model performance [75]. It also helps to mitigate the over-fitting issue in the model during the training stage. The overcast issue arises when random noise or mistakes occur instead of when the underlying connection is there. Using an increase in data, additional images were produced for the model from each picture because some irrelevant patterns may occur throughout the model training process. Several methods were employed for data augmentation operations: rotational changes, vertical and horizontal rotations, and intensity disorder, including light disturbances [76,77,78,79].

### 4.2. Deep Learning for Gesture Recognition

**Figure 7:**
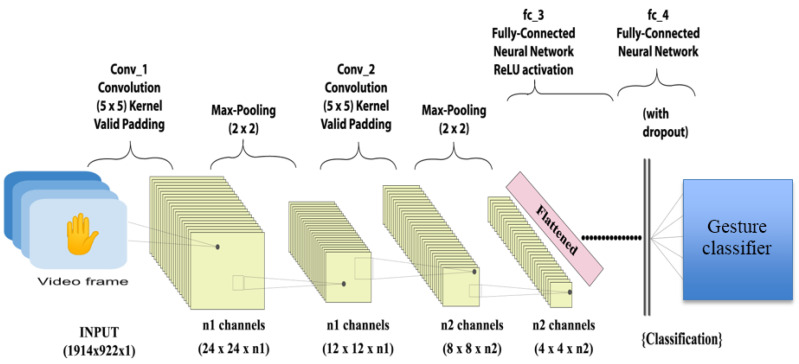
Gesture recognition using FCNN.

A classical ANN involves a local minimal issue, which typically ends with a local optimization process rather than a globally optimal state. More overfitting issues often complicates general machine learning models. Intensive network structure optimization may address the issues of the local minima and overriding by DNNs [80,81]. Deep learning is a machine learning-based approach that educates computers to accomplish tasks similar to those performed by humans. For example, deep learning is the underlying technology that enables driverless automobiles to detect traffic lights and people. It is also the underlying principle of audio and speech recognition in a variety of devices, such as mobile phones and tablets. Deep learning is gaining popularity because it is capable of performing tasks that were previously impossible. A deep learning model is constructed by layering data, which may be images, text, or audio, into distinct and discrete categorization layers. Artificial intelligence has the potential to provide findings that are 100 percent accurate with human-level precision and potentially beat humans in terms of speed. These models are developed using big data sets and machine learning approaches, such as CNN or ANN, both of which include several categorization layers. In machine learning approaches, the system would instruct the user on how to correctly utilize the model via the use of pictures, voice, and text. Deep learning models provide precise, accurate outcomes that are on par with human performance. These models are constructed using the data provided and turn them into artificial neural networks with many layers of categorized data. General data following the diagram of a fully convolutional neural network (FCNN) for gesture recognition are depicted below in Figure 7. Deep learning achieves unprecedented precision and accuracy, which enables it to match customer expectations. It is beneficial in a variety of applications, including autonomous vehicles. Recent advancements have shown that artificial intelligence is capable of outperforming humans in classifying photos. Deep learning requires a vast volume of labeled data. For example, building driverless vehicles requires the collection of hundreds of thousands of photos and movies. Deep learning needs an enormous amount of computational power. Elite GPUs are comparable in architecture and are well suited for deep learning. When cloud computing and clusters are integrated, it takes much less time than it did before, when it took weeks. Due to the fact that deep learning is based on neural networks, it is often referred to as “deep neural networks”. The term “deep” is often used to refer to the number of hidden layers in the neural system. Typically, neural networks comprise just 2–3 hidden layers; however, deep systems may contain up to 150 layers. They must train the deep learning models prior to implementing them. They need a vast quantity of labeled data and neural networks to train these models. This enables them to derive the characteristics directly from the data without requiring any human input.

One of the most well-known deep neural network methods is CNN. “Conventional neural network” is the abbreviation for “conventional neural network”. It employs 2D convolutional layers to handle 2D data and uses categorized layers of input data [82,83].

### 4.3. Summary

We give a review of current convolutional neural networks for action and gesture identification in image frames in this study. We provide a framework for handling both problems that covers key elements of deep learning as well as other hand-crafted methods. The suggested architectures, fusion methodologies, primary datasets, and competitions are discussed in depth. We outline and examine the key suggestions thus far, with a focus on how they deal with the data temporal component, suggesting potential and problems for future study based on 3D models [84,85,86,87,88,89,90,91,92]. Based on the evaluation, the associated difficulties and future research directions were discussed in the preceding section. It is critical to develop practical answers in the future. We think that the conversations in this section of the work will reveal fresh research gaps that will help us get closer to the much-desired next-generation technologies [93,94,95,96,97]. Hybrid models that combine handcrafted and new descriptors are predicted to advance. Similarly, we believe that deep learning solutions for large-scale, real-time action and gesture identification would be of interest to the community. In extended, uncut, and realistic videos, immediate effort is also anticipated in action/gesture localization. As a result, we anticipate that in the next years, emerging challenges, such as early recognition, multi-task learning, captioning, recognition from low resolution sequences, and life log devices will attract attention [97,98,99,100,101,102].

## 5. Conclusions

This paper provides a systematic review and analysis of recent vision-based gesture recognition methods in the design of more efficient and intelligent HCIs. In the area of vision-based hand gesture recognition, significant development has been achieved in the recent few years, both in terms of hardware and software. The evaluation results also show that the identification of hand gestures within the scientific community has created a great deal of attention among a broad range of techniques for recognizing vision-based gestures. Over the course of 11 years, this article looked at the difficulties and development of the vision-based hand gesture recognition system. Data gathering, features, and training environment seem to be covered in almost every article we investigated.

We have discussed the difficulties and challenges associated with gesture recognition in this article. We reviewed the most important algorithms used in gesture recognition as well. Hand gesture recognition is anticipated to play a significant part in our everyday lives in the modern world. The surrounding gadgets will almost certainly all have hand gesture interfaces sooner than one may imagine. The recognition of hand gestures is anticipated to play an important part in our daily lives. In the modern world, most of the technologies around us are mostly controlled by hand gestures. In the future, we want to improve our analytical approach to learn more about gesture recognition techniques.

## Figures and Tables

**Table 1:** Comparison of the most recent selected gesture recognition studies.

Author	Findings	Challenges
[56]	In this study, image processing techniques such as wavelets and empirical mode decomposition were suggested to extract picture functionalities in order to identify 2D or 3D manual motions. Classification of artificial neural networks (ANN), which was utilized for the training and classification of data in addition to the CNN (CNN).	Three-dimensional gesture disparities were measured utilizing the left and right 3D gesture videos.
[57]	Deaf–mute elderly folk use five distinct hand signals to seek a particular item, such as drink, food, toilet, assistance, and medication. Since older individuals cannot do anything independently, their requests were delivered to their smartphone.	Microsoft Kinect v2 sensor’s capability to extract hand movements in real time keeps this study in a restricted area.
[58]	The physical closeness of gestures and voices may be loosened slightly and utilized by individuals with unique abilities. It was always important to explore efficient human computer interaction (HCI) in developing new approaches and methodologies.	Many of the methods encounter difficulties like occlusions, changes in lighting, low resolution and a high frame rate.
[59]	A working prototype is created to perform gestures based on real-time interactions, comprising a wearable gesture detecting device with four pressure sensors and the appropriate computational framework.	The hardware design of the system has to be further simplified to make it more feasible. More research on the balance between system resilience and sensitivity is required.
[60]	This article offers a lightweight model based on the YOLO (You Look Only Once) v3 and the DarkNet-53 neural networks for gesture detection without further preprocessing, filtration of pictures and image improvement. Even in a complicated context the suggested model was very accurate, and even in low resolution image mode motions were effectively identified. Rate of high frame.	The primary challenge of this application for identification of gestures in real time is the classification and recognition of gestures. Hand recognition is a method used by several algorithms and ideas of diverse approaches for understanding the movement of a hand, such as picture and neural networks.
[61]	This work formulates the recognition of gestures as an irregular issue of sequence identification and aims to capture long-run spatial correlations in points of the cloud. In order to spread information from past to future while maintaining its spatial structure, a new and effective PointLSTM is suggested.	The underlying geometric structure and distance information for the object surfaces are accurately described in dot clouds as compared with RGB data, which offer additional indicators of gesture identification.
[62]	A new system is presented for a dynamic recognition of hand gestures utilizing various architectures to learn how to partition hands, local and global features and globalization and recognition features of the sequence.	To create an efficient system for recognition, hand segmentation, local representation of hand forms, global corporate configuration, and gesture sequence modeling need to be addressed.
[63]	This article detects and recognizes the gestures of the human hand using the method to classification for neural networks (CNN). This process flow includes hand area segmentation using mask image, finger segmentation, segmented finger image normalization and CNN classification finger identification.	SVM and the naive Bayes classification were used to recognize the conventional gesture technique and needed a large number of data for the identification of gesture patterns.
[64]	They provided a study of existing deep learning methodologies for action and gesture detection in picture sequences, as well as a taxonomy that outlines key components of deep learning for both tasks.	They looked through the suggested architectures, fusion methodologies, primary datasets, and competitions in depth. They described and analyzed the key works presented so far, focusing on how they deal with the temporal component of data and suggesting potential and challenges for future study.
[65]	They solve the problems by employing an end-to-end learning recurrent 3D convolutional neural network. They created a spatiotemporal transformer module with recurrent connections between surrounding time slices that can dynamically change a 3D feature map into a canonical view in both space and time.	The main challenge in egocentric vision gesture detection is the global camera motion created by the device wearer’s spontaneous head movement.
[66]	To categorize video sequences of hand motions, a long-term recurrent convolution network is utilized. Long-term recurrent convolution is the most common kind of long-term recurrent convolution. Multiple frames captured from a video sequence are fed into a network to conduct categorization in a network-based action classifier.	Apart from lowering the accuracy of the classifier, the inclusion of several frames increases the computing complexity of the system.
[67]	The MEMP network’s major characteristic is that it extracts and predicts the temporal and spatial feature information of gesture video numerous times, allowing for great accuracy. MEMP stands for multiple extraction and multiple prediction.	They present a neural network with an alternative fusion of 3D CNN and ConvLSTM since each kind of neural network structure has its own constraints. MEMP was developed by them.
[68]	This research introduces a new machine learning architecture that is especially built for gesture identification based on radio frequency. They are particularly interested in high-frequency (60 GHz) short-range radar sensing, such as Google’s Soli sensor.	The signal has certain unique characteristics, such as the ability to resolve motion at a very fine level and the ability to segment in range and velocity space rather than picture space. This allows for the identification of new sorts of inputs, but it makes the design of input recognition algorithms much more challenging.
[69]	They propose learning spatio-temporal properties from successive video frames using a 3D convolutional neural network (CNN). They test their method using recordings of robot-assisted suturing on a bench-top model from the JIGSAWS dataset, which is freely accessible.	Recognizing surgical gestures automatically is an important step in gaining a complete grasp of surgical expertise. Automatic skill evaluation, intra-operative monitoring of essential surgical processes, and semi-automation of surgical activities are all possible applications.
[70,71]	They blur the image frames from videos to remove the background noise. The photos are then converted to HSV color mode. They transform the picture to black-and-white format through dilation, erosion, filtering, and thresholding. Finally, hand movements are identified using SVM.	Gesture-based technology may assist the handicapped, as well as the general public, to maintain their safety and requirements. Due to the significant changeability of the properties of each motion with regard to various persons, gesture detection from video streams is a complicated matter.
[72,73]	The purpose of this study is to offer a method for Hajj applications that is based on a convolutional neural network model. They also created a technique for counting and then assessing crowd density. The model employs an architecture that recognizes each individual in the crowd, marks their head position with a bounding box, and counts them in their own unique dataset (HAJJ-Crowd).	There has been a growth in interest in the improvement of video analytics and visual monitoring to better the safety and security of pilgrims while in Makkah. It is mostly due to the fact that Hajj is a one-of-a-kind event with hundreds of thousands of people crowded into a small area.
[74]	This study presents crowd density analysis using machine learning. The primary goal of this model is to find the best machine learning method for crowd density categorization with the greatest performance.	Crowd control is essential for ensuring crowd safety. Crowd monitoring is an efficient method of observing, controlling, and comprehending crowd behavior.

## Data Availability

Not applicable.
